# ADDUCTOR POLLICIS MUSCLE THICKNESS AND PREDICTION OF POSTOPERATIVE MORTALITY IN PATIENTS WITH STOMACH CANCER

**DOI:** 10.1590/0102-672020180001e1340

**Published:** 2018-03-01

**Authors:** Aline Kirjner POZIOMYCK, Oly Campos CORLETA, Leandro Totti CAVAZZOLA, Antonio Carlos WESTON, Edson Braga LAMEU, Luisa Jussara COELHO, Luis Fernando MOREIRA

**Affiliations:** 1 Program of Post-Graduation in Surgical Sciences. Federal University of Rio Grande do Sul - UFRGS. Porto Alegre. RS; 2 Department of Digestive Surgery. Lutheran University of Brazil - ULBRA. Canoas. RS; 3 Department of Nutrology. Federal University of Rio de Janeiro - UFRJ. Rio de Janeiro. RJ; 4 Program of Post-Graduation in Epidemiology. Federal University of Rio Grande do Sul - UFRGS. Porto Alegre. RS. Brazil

**Keywords:** Stomach neoplasms, Nutrition assessment, Malnutrition, Mortality, Neoplasias gástricas, Avaliação nutricional, Desnutrição, Mortalidade

## Abstract

**Background:**

Malnutrition is very prevalent in patients with gastric cancer and increases the risk of morbidity and mortality. Adductor pollicis muscle thickness (APMT) appears as an important objective, quick, inexpensive and noninvasive measure to assess the muscle compartment

**Aim:**

To compare APMT and other nutritional assessment methods and to correlate these methods with postoperative mortality

**Methods:**

Forty-four patients, 29 men and 15 women, mean age of 63±10.2 and ranging from 34-83 years, who underwent nine (20.5%) partial and 34 (77.3%) total gastrectomies due to stomach cancer (stage II to IIIa) were preoperatively assessed by Patient Generated Subjective Global Assessment (PG-SGA), anthropometry and laboratorial profile

**Results:**

APMT better predicted death (p<0.001) on both, dominant and non-dominant hand, and well correlated with albumin (p=0.039) and PG-SGA (p=0.007)

**Conclusion:**

APMT clearly allowed to determine malnutrition and to predict risk of death in patients with gastric cancer.

## RESUMO

**Racional:**

A desnutrição é muito prevalente em pacientes com câncer gástrico e aumenta o risco de morbidade e mortalidade. A espessura do músculo adutor do polegar (APMT) aparece como uma importante medida objetiva, rápida, barata e não invasiva para avaliar o compartimento muscular.

**Objetivo:**

O objetivo deste estudo foi comparar a APMT e outros métodos de avaliação nutricional e correlacionar esses métodos com a mortalidade pós-operatória.

**Métodos:**

Quarenta e quatro pacientes, 29 homens e 15 mulheres; média (SD) de 63 anos (10,2) e variando de 34 a 83 anos, que foram submetidos a 9 (20,5%) gastrectomias parciais e 34 (77,3%) totais por câncer de estômago (Estágio II a IIIa) e avaliados no pré operatório por Avaliação Subjetiva Global Produzida Pelo Paciente (PG-SGA), antropometria e perfil laboratorial.

**Resultados:**

APMT melhor predisse morte (p<0,001) em ambas mãos, dominante e não-dominante, e se correlacionou bem com albumina (p=0,039) e PG-SGA (p=0,007).

**Conclusão:**

APMT permitiu claramente determinar a desnutrição e prever o risco de morte em pacientes com câncer gástrico.

## INTRODUCTION

Cancer has been currently considered as a major public health problem worldwide^28^, and malnutrition is a major cause of morbidity and mortality^28-30.^ Incidence of malnutrition in cancer patients ranges from 40-80% and patients with gastrointestinal tract cancer have a significant greater loss than those with tumors of other sites^30^. Despite high prevalence, approximately 50% malnourished patients are not well recognized by the medical staff at admission and therefore are not properly treated^3^.

Malnourished patients are less likely to tolerate oncologic treatment. including surgery, than well-nourished ones. Nutritional depletion is a common problem in patients with cancer and is associated with poorer outcome^26^ and more serious complications such as poor wound healing, fistula formation, infection as well as increased length of hospital stay and hospital costs, decreased survival and quality of life^30^.

Malnutrition is defined as a deficiency status of macronutrients, micronutrients and energy that can produce considerable changes on body functions^27^. Cancer patients often have a decreased food intake due to a number of causes direct related to cancer *per se* and some authors suggested that 20% of deaths are related to malnutrition and not only due to cancer itself^30^.

By early evaluation of nutritional status and by restoring depleted nutrients, it can be minimized or virtually eliminated morbidity or mortality related to malnutrition^13^.

There is still no acceptable consensus in clinical practice as to the gold standard on which diagnostic tool can allow adequate identification of malnutrition in adults^25^. All currently assessed parameters can be affected by a number of diseases and injuries. Moreover, it is difficult to distinguish malnutrition effects of those resulting from tumor progression, as well as it remains unclear which method commonly used would be more reliable due to lack of comparative data^20^.

Among the conventional anthropometric measurements, evaluation of adductor pollicis muscle thickness (APMT) appears as an important variable of objective measure to assess muscle compartment, being considered a quick, inexpensive and noninvasive method^29^. Patient-Generated Subjective Global Assessment (PG-SGA) has been used as a gold standard in many studies. However, being a subjective assessment proper understanding and good performance is crucial since its accuracy depends on the observer’s ability. Other methods more or less expensive and feasible are also used in clinical practice. A combination of different methods is still the preferred choice of most professionals^25^.

In a previous study, concerning nutritional assessment in patients with gastrointestinal tract tumors, APMT demonstrated association with malnourishment^25^. Therefore, the aim of this study was to compare and correlate APMT with other nutritional assessment methods to determine its power in predicting postoperative mortality. 

## METHODS

This is a prospective study, including 44 patients (29 men and 15 women), mean age of 63±10.2 years, ranging from 34-83, who underwent gastrectomy due to gastric cancer at Santa Rita Cancer Hospital, Santa Casa de Misericórdia Center, Porto Alegre, RS, Brazil, from March 2010 to March 2012. This project was carried out by the Southern Surgical Oncology Research Group (SSORG), and was approved by the Institutional Review Board of the Santa Casa Hospital Facilities under IRB#2041/08.

Patients older than 18 years with a histologically proven gastric adenocarcinoma who underwent gastrectomy and agreed to sign the written consent, were included.

To determine the sample size, it was considered based on the relationship between DAPMT and death, where the difference average DAPMT between groups died (10.8±3.7) and survived (13.9±2.9) was approximately 30%. Thus, assuming an error margin of 13% to achieve the estimated difference, and a significance level of 5% (a=0.05) and 80% sample power (1-ß), the minimum estimated size sample was 39 patients. As this study considered the convenience sampling, were included 44 patients who agreed to participate in the previously established period.

Patients were assessed by the same protocol within 72 h of hospital admission by the PG-SGA. classical anthropometry including current weight, percentage of weight loss (%WL); body mass index (BMI), arm circumference (AC) and mid-arm muscle circumference (MAMC), triceps skinfold (TSF), adductor pollicis muscle thickness (APMT) and laboratory measurements of albumin and total lymphocyte count (TLC). 

In this study was used an adapted Portuguese validated version of PG-SGA, proposed to assess oncologic patients^19^ and the results were described into three categories as A (well nourished), B (risk of malnutrition or moderate malnourished) and C (severely malnourished). The sum of the scores was used to determine specific nutritional approaches^23^.

To measure current weight and height was used a digital scale Welmy^®^ platform type graduated in 100 g and a measuring ruler previously calibrated in the same scale in which the weight was measured. Were used the tables proposed by Lipschitz et al.^18^ and WHO^21^ to classify BMI of adult and elderly patients, respectively. The usual weight was recorded as referred by the patients, and it was considered to calculate the percentage of weight loss^5^. AC was measured using a non-extendable plastic tape in the non-dominant arm and MAMC was calculated according to Frisancho^9^. TSF was measured by a Lange^®^ caliper with accuracy of 1mmand the pressure of the spring is constant at 10 g/mm². APMT was measured with patient in a sitting position, hands lying on knees and elbow at an angle of approximately 90° over the ipsilateral lower limb. The Lange^®^ caliper was used,using a continuous pressure of 10 g/mm^2^ to pinch the adductor muscle in the vertex of an imaginary triangle comprised by the extension of the thumb and index finger^15^ ^,^ ^16^. The common used hand for writing or physical activity was considered as the dominant one. The average of three distinct measurements was considered to be the mean APMT. All measurements were performed by the same observer (AKP) and checked in triplicate attempting to reduce biases. Laboratory profile was obtained through routine blood samples from all patients.

### Statistical analysis

Included the Pearson, chi-square with continuity correction or Fisher exact test by Monte Carlo simulation. Were used for quantitative approach, Kolmogorov-Smirnov tests; *t*-student test for independent groups; Mann-Whitney and Pearson correlation. SPSS (Statistical Package to Social Sciences for Windows) 17.0 was used for all analyses and a p level of 5% was considered to be significant.

## RESULTS

Overall a total of 44 patients (29 men, 15 women) were included in the study. All patients were diagnosed as AJCC 2010 pathological stage II or III. All but one underwent gastrectomy, being nine (20%) partial and 34 (77%) total gastrectomies. The remaining patient was submitted to esophagogastrectomy.

Dominant hand of adductor pollicis muscle (DAPMT), non-dominant hand of adductor pollicis muscle (NDAPMT) and PG-SGA had the better prediction of 30-day postoperative death (Table 1).

**TABLE 1:** Mortality in 30 days of gastric cancer patients

	30-days death (n)		p
	Yes (6)	No (38)	
	Mean SD	Mean SD	
DAPMT	8.2 1.9	14.1 3.4	<0.001*
NDAPMT	7.6 1.9	13.9 5.7	0.001*
Albumin	3.4 0.8	3.9 0.4	0.026*
TLC	1414.8 553.1	1486.8 737.3	0.497+
PG-SGA			
A	0 (0.0%)	15 (39.5%)	
B	0 (0.0%)	18 (47.4%)	<0.001+
C	6 (100.0%)	5 (13.2%)	

DAPMT=dominant adductor pollicis muscle thickness; NDAPMT=non-dominant adductor pollicis muscle thickness; TLC=total lymphocyte count; PG-SGA=patient generated subjective global assessment; * t-student test; + Mann-Whitney test; + Fisher’s exact test by Monte Carlo simulation.

Correlation of APMT with other nutritional parameters is presented in Table 2. A significant moderately negative correlation of DAPMT (r=-0.399; p=0.007) and NDAPMT (r=-0.372; p=0.013) with PG-SGA was observed. Comparing DAPMT with albumin, a significant moderately positive correlation was demonstrated (r=0.319; p=0.039), indicating that the higher the DAPMT the greater the levels of albumin.

**TABLE 2:** Correlation of APMT with other nutritional parameters

Nutritional parameters		DAPMT (mm)	NDAPMT (mm)	Albumin (g/dl)	TLC (mm^3^)	BMI (kg/m^2^)
DAPMT	Correlation coefficient					
	Sig. (2-tailed)					
	n					
NDAPMT	Correlation coefficient	0.922+				
	Sig. (2-tailed)	0.000				
	n	44				
ALBUMIN	Correlation coefficient	0.319^*^	0.296			
	Sig. (2-tailed)	0.039	0.057			
	n	42	42			
TLC	Correlation coefficient	0.286	0.224	0.391^*^		
	Sig. (2-tailed)	0.060	0.143	0.010		
	n	44	44	42		
BMI	Correlation coefficient	0.130	0.145	0.210	-0.069	
	Sig. (2-tailed)	0.401	0.346	0.183	0.655	
	n	44	44	42	44	
PG-SGA	Correlation coefficient	-0.399+	-0.372^*^	-0.552+	-0.312^*^	-0.311^*^
	Sig. (2-tailed)	0.007	0.013	0.000	0.039	0.040
	n	44	44	42	44	44

APMT=adductor pollicis muscle thickness; DAPMT=dominant adductor pollicis muscle thickness; NDAPMT=non-dominant adductor pollicis muscle thickness; TLC=total lymphocyte count; BMI=body mass index; PG-SGA=patient generated subjective global assessment; Spearman´s rho correlations, Correlation coefficient:* 5% of significance */+ 1% of significance.

Table 3 shows a statistically significant association of death when patient is assessed as B or C according to the PG-SGA or when the APMT measures are the worst.

**TABLE 3:** Measures for DAPMT and NDAMPT according to PG-SGA and mortality

Death			n	Mean	SD	95% CI for mean	p
Yes	DAPMT (mm)	A	1	16.33	-		
		B	6	13.39	3.41	9.81 - 16.97	0.012
		C	6	8.22	1.91	6.22 - 10.22	
	NDAPMT (mm)	A	1	13.00	-		
		B	6	12.67	3.44	9.05 - 16.28	0.025
		C	6	7.56	1.96	5.50 - 9.62	
No	DAPMT (mm)	A	14	14.52	3.68	12.40 - 16.65	
		B	12	13.92	3.27	11.84 - 15.99	0.706
		C	5	13.00	3.84	8.24 - 17.76	
	NDAPMT (mm)	A	14	15.60	8.25	10.83 - 20.36	
		B	12	12.94	3.51	10.72 - 15.17	0.475
		C	5	12.53	4.20	7.32 - 17.75	

DAPMT=dominant adductor pollicis muscle thickness; NDAPMT=non-dominant adductor pollicis muscle thickness; PG-SGA=patient generated subjective global assessment

There were significant statistical differences of DAPMT (p=0.023) and NDAPMT (p=0.049) in relation to death. In both cases the mean of APMT in patients who died were significantly lower. Nutritional parameters and hospital stay, as well as their correlation with AMPT, are presented in Table 4.

**TABLE 4:** Nutritional parameters and its correlations with DAPMT and NDAPMT

Variables (N=44)	Mean	SD	Median	95%CI	Correlation (r) with APMT	
					DAPMT	NDAPMT
Age (years)	61.5	10.8	62.0	59.2-65.6	-0.071	0.024
Length of stay (days)	25.4	23.2	17.0	17.7-32.6	-0.050	-0.128
% Weight loss	12.7	9.8	11.3	9.3-15.3	-0.232	-0.137
Body mass index (kg/m2)	23.3	4.1	23.2	21.8-24.5	0.178	0.204
Triceps skinfold (mm)	13.3	6.3	13.0	10.8-14.8	0.134	0.333*
Mid-arm muscle circumference (cm)	24.0	4.3	23.6	22.7-25.4	0.327*	0.091
Albumin (g/dl)	3.9	0.5	3.9	3.7-4.0	0.495+	0.314*
TLC (mm3)	1476.8	710.0	1386.7	1393.7-1775.8	0.264	0.310*
DAPMT (mm)	13.2	3.8	13.2	12.1-14.5	-	-
NDAPMT (mm)	13.0	5.8	12.2	11.1-14.8	-	-
PG-SGA	-	-	-		-0.399*	-0.372*

APMT=adductor pollicis muscle thickness; DAPMT=dominant adductor pollicis muscle thickness; NDAPMT=non-dominant adductor pollicis muscle thickness; TLC=total lymphocyte count; PG-SGA=patient generated subjective global assessment; Correlation coefficient: * 5% of significance */+ 1% of significance; 95%CI=confidence interval of 95%.

## DISCUSSION

Nutritional assessment performed immediately following admission allows establishment of an early diet therapy plan in order to improve nutritional status and to minimize the risk of postoperative complications.

The APMT is the only muscle that allows directly evaluation and measure of thickness since is located between two bones and has a definite anatomical location^15^ ^,^ ^16^. Furthermore, the APMT is easily accessible and is minimally affected by adjacent subcutaneous fat tissue^4^.

Gonzalez et al.^11^ evaluating healthy subjects observed a significant association only between measured AMPT and BMI and Oliveira^22^ showed a significant association of lean mass (p<0.005) and handgrip (p=0.0024) in stroke patients, while APMT was well correlated with anthropometry and bioimpedance. On kidney dysfunction, Oliveira et al.^8^ observed a significant association of AMPT with BMI, phase angle, albumin and mid-arm muscle circumference, while Pereira et al.^24^ positively correlated APMT with handgrip (p<0.05), serum albumin (p=0.07) and inversely with age even when linear regression analysis was adjusted for sex, age and time on hemodialysis.

Our results demonstrated that nutritional status based on AMPT had a good correlation with other nutritional parameters such as the PG-SGA that is considered as a gold standard method in many studies^25^. APMT also had a good prediction of 30-day mortality considered as a good outcome in clinical investigations. The median of APMT found is similar to that presented by Valente et al.^29^ in patients submitted to surgical procedures, including those ones with cancer as well.

There are few published studies assessing APMT^12^ ^,^ ^19^ ^,^ ^25^ ^,^ ^29^ and its relation with other nutritional parameters predicting both, mortality and length of stay. Distinct cutoffs have been suggested from different studies in specific clinical condition such as intensive care^7^ ^,^ ^10^ ^,^ ^14^ ^,^ ^17^, breast cancer^2^, kidney dysfunction^24^, hemorrhagic stroke^22^ and general gastrointestinal surgical procedures^6^ ^,^ ^19^. Even for healthy adult population there are no reliable and established range and average figures to be used as a parameter of normal range^11^ ^,^ ^15^ ^-^ ^16^. To date, to our knowledge, there is no study testing AMPT assessment in patients undergoing gastrointestinal cancer surgical procedures to provide a reliable comparison of results as assessed in this study.

 Ghorabi et al.^10^ by prospectively evaluating 127 ICU patients, 55 (43%) of them who were surgical patients, found in the modified multivariate regression analysis. APMT had higher correlation with mortality (OR=5.6; p<0.001) and length of stay >10 days (OR=11.3; p<0.001). On evaluating 248 patients admitted either to a medical or surgical ICU, Caporossi et al.^7^ found APMT significantly lower (p<0.001) in severe unnourished patients as compared to nourished ones; and risk of death was 6-fold higher in subjects with thinner APMT (OR=6.3; p=0.02). Nevertheless, no correlation with length of stay and mechanical ventilation time were found. ROC curves between APMT and SGA APMT showed accuracy with an AUC of 0.82, though neither death or length of stay were statistically significant in a cross-sectional study with 83 heart intensive care patients^14^. In contrast, a study conducted in Singapure^17^ with 229 patients in ICU (no diagnosis specified). APMT failed to show significance for mortality at 28 days or for length of stay.

On surgical patients, mid-arm muscle circumference (MAMC), commonly referred as a good parameter for muscle load showed a good correlation with APMT (p<0.05), unlikely what was observed by Melo et al.^19^ with 151 patients scheduled for elective surgical procedure (40% with gastrointestinal cancer) that found association with AC (p=0.007), TSF (p=0.001) and BMI (p=0.001).

In a study with 150 candidates for reconstructive surgery, Valente et al.^29^ found a significant association between APMT and SGA (p=0.021) and BMI (p=0.008) for nutritional risk. Also a significant correlation was demonstrated between APMT and MAMC (R^2^=0.326), corrected arm muscle area (R^2^=0.371) and BMI (R^2^=0.290). Braganolo et al.^6^ also showed a good association (p<0.05) between AMPT and other nutritional assessment methods such as BMI, %WL, TSF and MAMC. There were no association with laboratory profile and APMT mean values in eutrophic patients by SGA significantly higher (p<0.001) than less unnourished, and these were also higher (p<0.05) than severe malnutrition (ASG-C). In this study these parameters, %WL and BMI did not correlate significantly with AMPT.

Gonzalez et al.^12^ studying surgical patients (n=361), none with cancer found a direct correlation (-0.61, p<0.05) between SGA and DAMPT showing significantly higher values of AMPT in well nourished patients assessed by SGA, as seen also in this study.

The use of APMT proved to be an adequate method to detect malnutrition in surgical patients in many studies^6^ ^,^ ^12^ ^,^ ^19^ ^,^ ^27^ and it should be added to the screening process for inpatients, since it is easy to perform, low-cost and noninvasive^29^.

Worldwide studies are needed to compare results and to consider multiracial land local differences that can significantly affect APMT measurement, especially in countries such as Brazil, where ethnicity and race are usually self-attributed and may hinder accuracy for estimates. An ongoing study shall better determine an APMT cutoff measure predictable of poor outcome. 

## CONCLUSION

APMT measurement is adequate to determine nutrition status in patients with gastric cancers. Correlates well with early postoperative death and should be considered for routine of nutritional status. 

## References

[1] Andreoli A, De Lorenzo A, Cadeddu F, Iacopino L, Grande M (2011). New trends in nutritional status assessment of cancer
patients. Eur Rev Med Pharmacol Sci.

[2] Bering T, Mauricio SF, Silva JB, Correia MI (2014). Nutritional and metabolic status of breast cancer
women. Nutr Hosp.

[3] Bertuccio P, Chatenoud L, Levi F, Praud D, Ferlay J, Negri E (2009). Recent patterns in gastric cancer: a global
overview. Int J Cancer.

[4] Bielemann RM, Horta BL, Orlandi SP, Barbosa-Silva TG, Gonzalez MC, Assuncao MC (2015). Is adductor pollicis muscle thickness a good predictor of lean
mass in adults. Clin Nutr.

[5] Blackburn GL, Bistrian BR, Maini BS, Schlamm HT, Smith MF (1977). Nutritional and metabolic assessment of the hospitalized
patient. JPEN J Parenter Enteral Nutr.

[6] Bragagnolo R, Caporossi FS, Dock-Nascimento DB, de Aguilar-Nascimento JE (2009). [Adductor pollicis muscle thickness: a fast and reliable method
for nutritional assessment in surgical patients]. Rev Col Bras Cir.

[7] Caporossi FS, Caporossi C, Borges Dock-Nascimento D, de Aguilar-Nascimento JE (2012). Measurement of the thickness of the adductor pollicis muscle as a
predictor of outcome in critically ill patients. Nutr Hosp.

[8] de Oliveira CM, Kubrusly M, Mota RS, Choukroun G, B J, da Silva CA (2012). Adductor pollicis muscle thickness: a promising anthropometric
parameter for patients with chronic renal failure. J Ren Nutr.

[9] Frisancho AR (1984). New standards of weight and body composition by frame size and
height for assessment of nutritional status of adults and the
elderly. Am J Clin Nutr.

[10] Ghorabi S, Ardehali H, Amiri Z, Vahdat Shariatpanahi Z (2016). Association of the Adductor Pollicis Muscle Thickness With
Clinical Outcomes in Intensive Care Unit Patients. Nutr Clin Pract.

[11] Gonzalez MC, Duarte RR, Budziareck MB (2010). Adductor pollicis muscle: reference values of its thickness in a
healthy population. Clin Nutr.

[12] Gonzalez MC, Pureza Duarte RR, Orlandi SP, Bielemann RM, Barbosa-Silva TG (2015). Adductor pollicis muscle: A study about its use as a nutritional
parameter in surgical patients. Clin Nutr.

[13] (2002). Guidelines for the use of parenteral and enteral nutrition in
adult and pediatric patients. JPEN J Parenter Enteral Nutr.

[14] Karst FP, Vieira RM, Barbiero S (2015). Relationship between adductor pollicis muscle thickness and
subjective global assessment in a cardiac intensive care
unit. Rev Bras Ter Intensiva.

[15] Lameu EB, Gerude MF, Campos AC, Luiz RR (2004). The thickness of the adductor pollicis muscle reflects the muscle
compartment and may be used as a new anthropometric parameter for
nutritional assessment. Curr Opin Clin Nutr Metab Care.

[16] Lameu EB, Gerude MF, Correa RC, Lima KA (2004). Adductor pollicis muscle: a new anthropometric
parameter. Rev Hosp Clin Fac Med Sao Paulo.

[17] Leong Shu-Fen C, Ong V, Kowitlawakul Y, Ling TA, Mukhopadhyay A, Henry J (2015). The adductor pollicis muscle: a poor predictor of clinical
outcome in ICU patients. Asia Pac J Clin Nutr.

[18] Lipschitz DA (1994). Screening for nutritional status in the elderly. Prim Care.

[19] Melo CY, Silva SA (2014). Adductor pollicis muscle as predictor of malnutrition in surgical
patients. Arq Bras Cir Dig.

[20] Nelson KA (2000). The cancer anorexia-cachexia syndrome. Semin Oncol.

[21] (2000). Obesity: preventing and managing the global epidemic. Report of a
WHO consultation. World Health Organ Tech Rep Ser.

[22] Oliveira DR, Frangella VS (2010). [Adductor pollicis muscle and hand grip strength: potential
methods of nutritional assessment in outpatients with
stroke]. Einstein (Sao Paulo).

[23] Ottery F (1996). Definition of standardized nutritional assessment and
interventional pathways in oncology. Nutrition.

[24] Pereira RA, Caetano AL, Cuppari L, Kamimura MA (2013). Adductor pollicis muscle thickness as a predictor of handgrip
strength in hemodialysis patients. J Bras Nefrol.

[25] Poziomyck AK, Weston AC, Lameu EB, Cassol OS, Coelho LJ, Moreira LF (2012). Preoperative nutritional assessment and prognosis in patients
with foregut tumors. Nutr Cancer.

[26] Ryu SW, Kim IH (2010). Comparison of different nutritional assessments in detecting
malnutrition among gastric cancer patients. World J Gastroenterol.

[27] Shim H, Cheong JH, Lee KY, Lee H, Lee JG, Noh SH (2013). Perioperative nutritional status changes in gastrointestinal
cancer patients. Yonsei Med J.

[28] Torre LA, Bray F, Siegel RL, Ferlay J, Lortet-Tieulent J, Jemal A (2015). Global cancer statistics. CA Cancer J Clin.

[29] Valente KP, Silva NM, Faioli AB, Barreto MA, Moraes RA, Guandalini VR (2016). Thickness of the adductor pollicis muscle in nutritional
assessment of surgical patients. Einstein (Sao Paulo).

[30] Waitzberg DC. WT, Correia MI (2001). Hospital malnutrition: the Brazilian national survey (IBRANUTRI):
a study of 4000 patients. Nutrition.

